# Supplementary figures and images for: Characterizing the variation in chromosome structure ensembles in the context of the nuclear microenvironment
Source: PLoS Comput Biol. 2022 Aug 15;18(8):e1010392. doi: 10.1371/journal.pcbi.1010392 (PMC9410561; doi:10.1371/journal.pcbi.1010392)

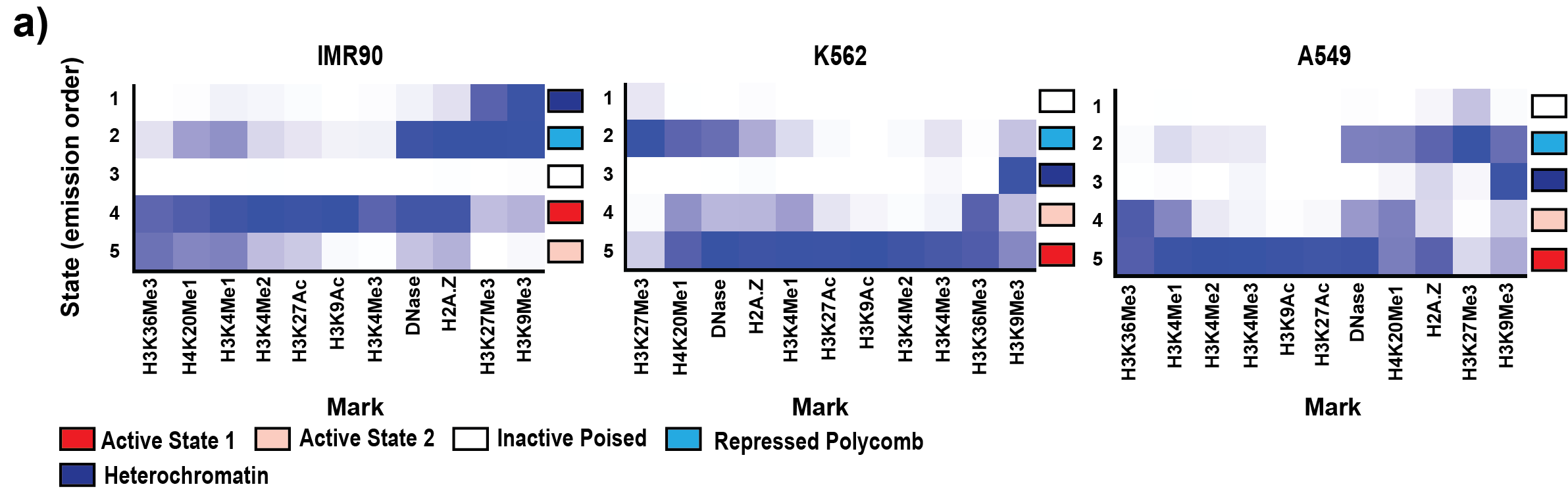

Supplement: S1 Fig — Assignment of different chromatin states based on relative enrichment of different histone marks using ChromHMM tool for chr21:28–30 Mb genomic region from three different cell-types—IMR90, K562 and A549 at 30 Kb resolution. (PNG) [file pcbi.1010392.s003.png]

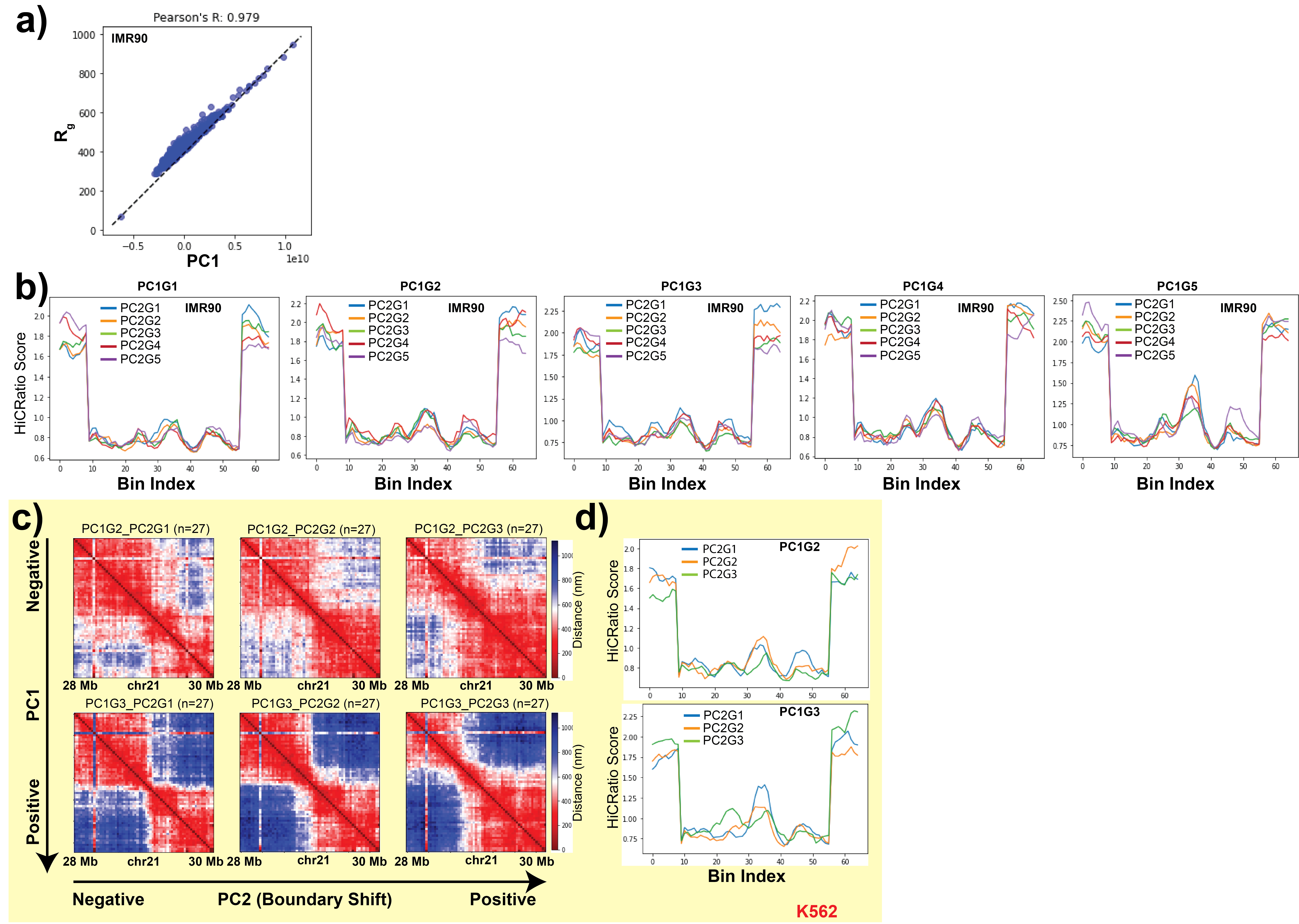

Supplement: S2 Fig — a) Pearson’s correlation between chromosome structural landscape PC1 values and radius of gyration (Rg) of single-cell structures (calculated from 3D position traces) for IMR90 chr21:28–30 Mb genomic region. b) HiCRatio score of PC2 ordered structures belonging to different PC1 groups for IMR90 chr21:28–30 Mb genomic region, calculated by HiCRatio approach (with a 300 kb window size). Here, PC1G1 refers to the group1 (G1; smallest Rg structures) based on PC1. Within PC1G1, then PC2G1 means the group1 (G1) based on PC2. Successive higher numbered PC1 groups have higher Rg structures. c) For K562 chr21:28–30 Mb genomic region, structures are first divided into three groups based on the PC1 ordering. Within each group, structures are further divided into three subgroups based on their PC2 ordering. To display the domain reorganization along PC2 in K562, averaged distance maps from only groups 2 and 3 from PC1 are shown here. d) For the same groups as in c, HiCRatio (with a 300 kb window size) scores of structures are shown. Colored lines represent HiCRatio scores from PC2 subgroups within two different PC1 subgroups (top = smaller Rg and bottom = larger Rg) (PNG) [file pcbi.1010392.s004.png]

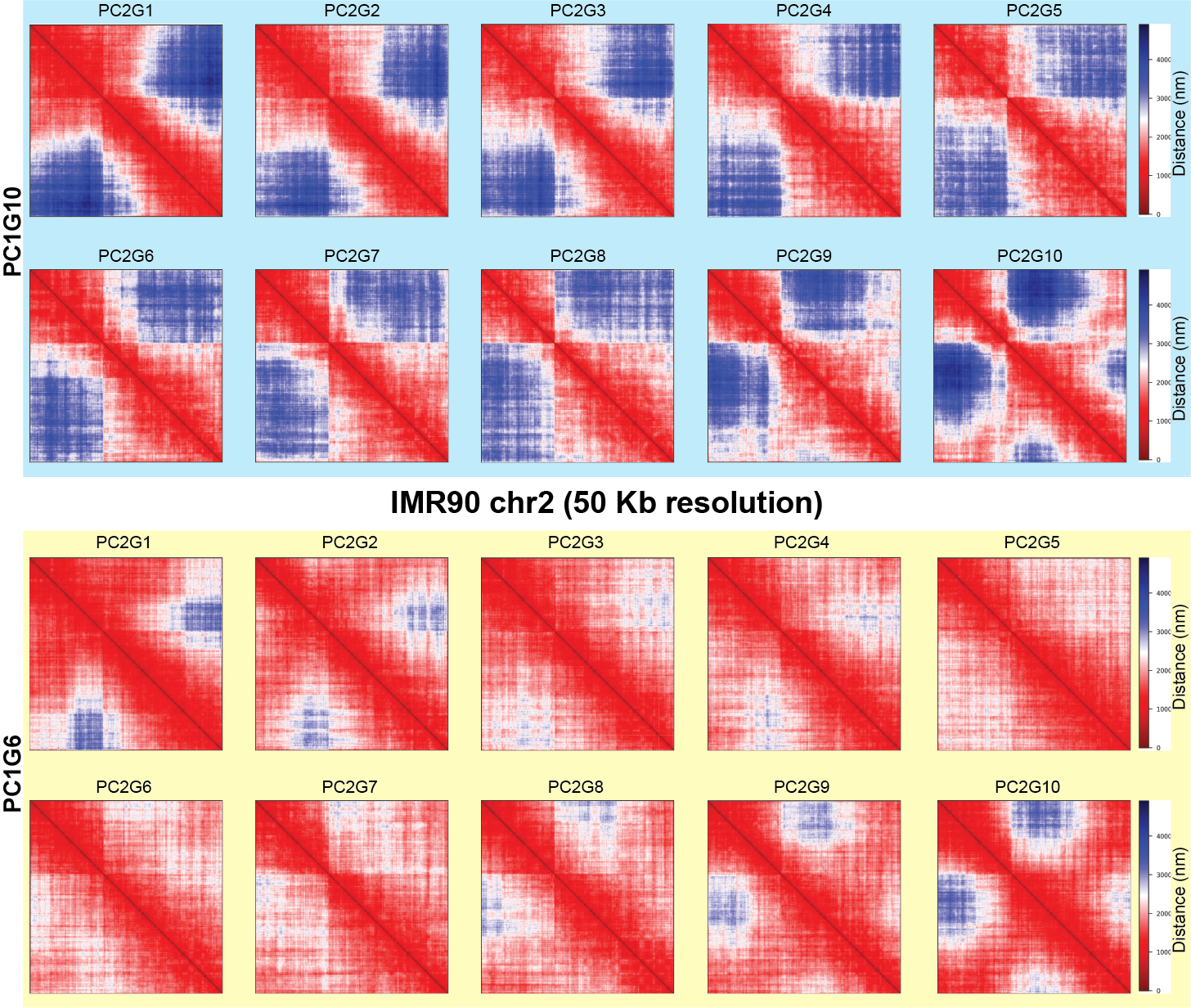

Supplement: S3 Fig — For IMR90 chr2 structure data, structures are first divided into ten groups based on the PC1 ordering. Within each group, structures are further divided into ten subgroups based on their PC2 ordering. To display the domain reorganization phenomenon along PC2 in IMR90 chr2, only PC1 subgroups 6 (lower Rg) and 10 (higher Rg) are shown here. Heatmaps display the average distances for structures within each PC2 subgroup in each PC1 subgroup. (PNG) [file pcbi.1010392.s005.png]

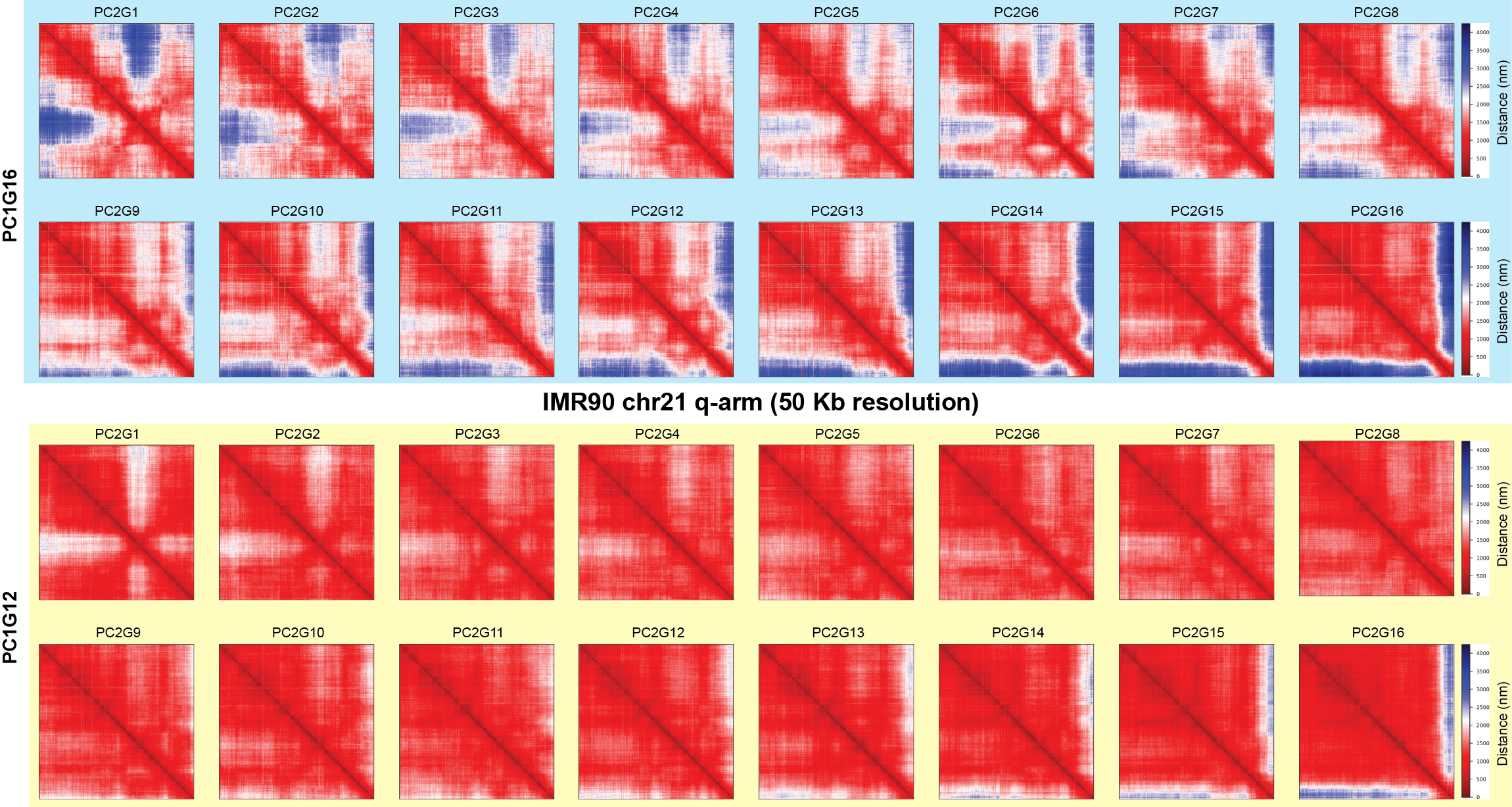

Supplement: S4 Fig — For IMR90 chr21 entire q-arm structure data, structures are first divided into sixteen groups based on the PC1 ordering. Within each group, structures are further divided into sixteen subgroups based on their PC2 ordering. To display the domain reorganization phenomenon along PC2 in IMR90 chr21, only PC1 subgroups 12 (lower Rg) and 16 (higher Rg) are shown here. Heatmaps display the average distances for structures within each PC2 subgroup in each PC1 subgroup. (PNG) [file pcbi.1010392.s006.png]

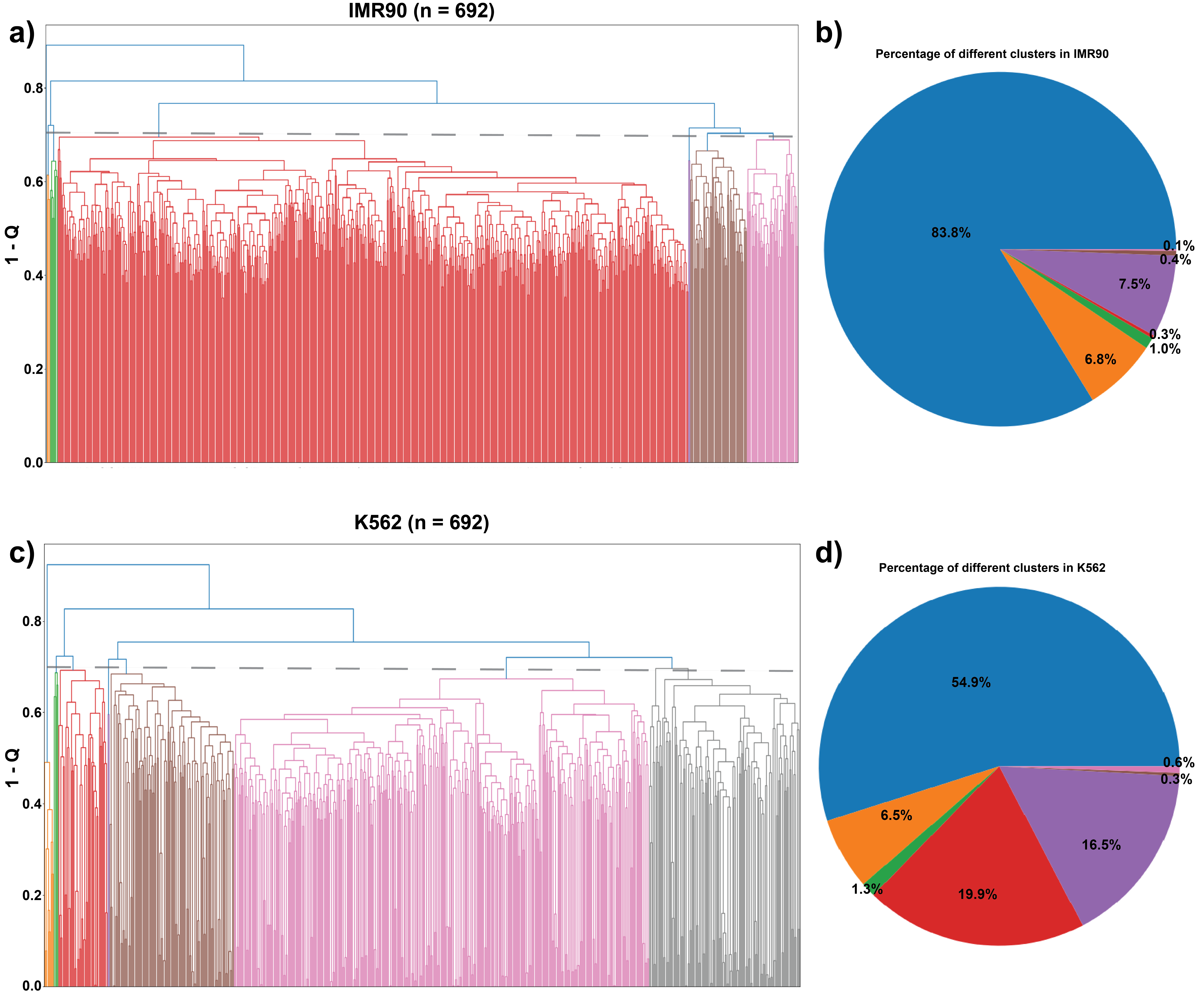

Supplement: S5 Fig — a,c) Hierarchical clustering of IMR90 (a) and K562 (c) structures for chr21:28–29.5 Mb genomic segment, based on Q similarity metric as described in Cheng et al. (1). To assign structures to the different clusters, the trees are cut at 0.7. b,d) Proportion of structures in different clusters obtained from IMR90 (b) and K562 (d) structural ensembles. (PNG) [file pcbi.1010392.s007.png]

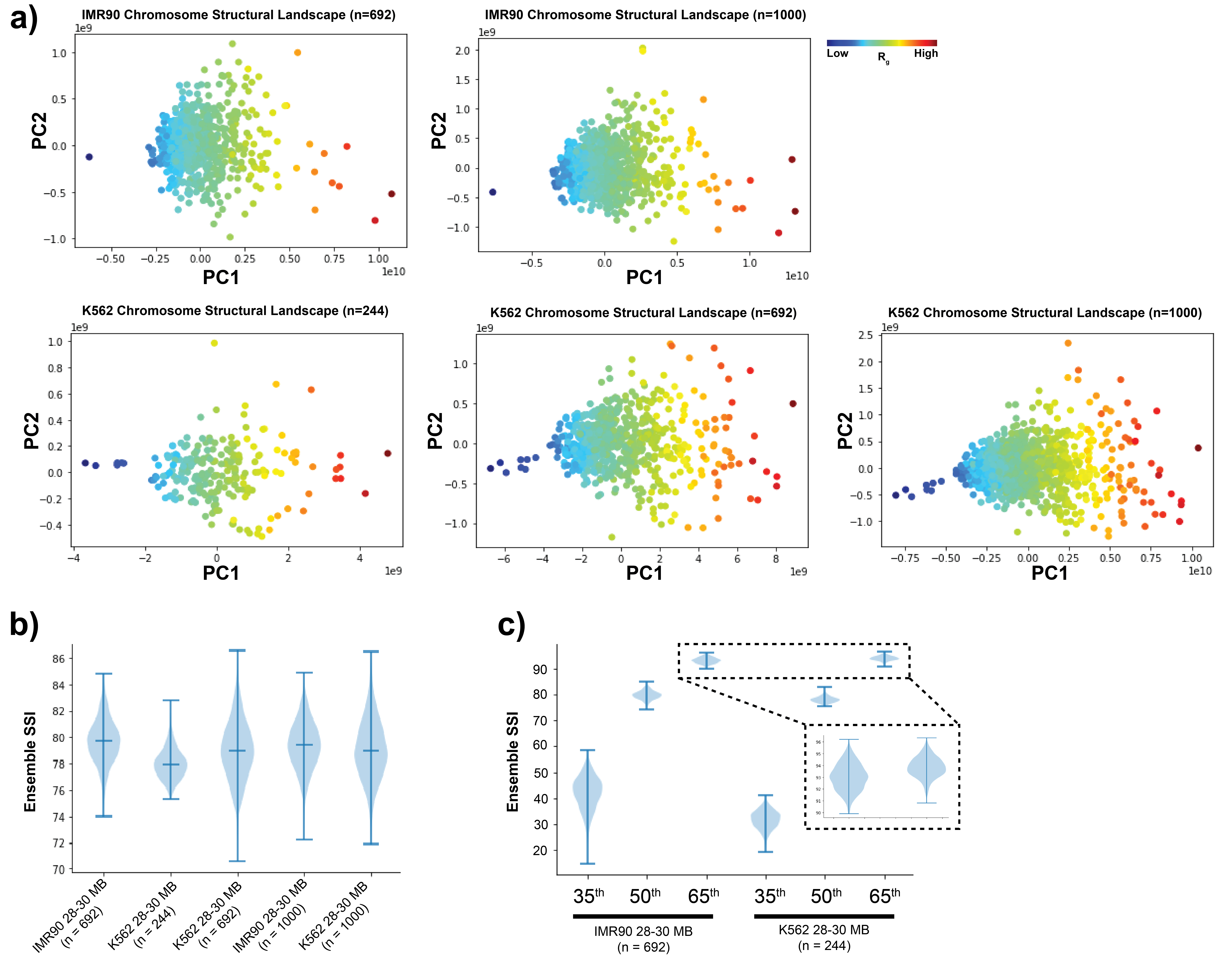

Supplement: S6 Fig — a) Chromosome structural landscape of IMR90 and K562 chr21:28–30 Mb chromatin regions constructed for different numbers of cells. Points are colored according to the radius of gyration (Rg) of the corresponding structures (dark blue–smaller Rg and dark red–larger Rg). b) Chromosome structural ensemble SSI distributions for chr21:28–30 Mb segment from IMR90 and K562 for different number of cells (shown as n). c) Chromosome structural ensemble SSI distributions for the same chr21:28–30 Mb segment from IMR90 and K562 for a lower (35th percentile) and higher (65th percentile) correlation matrix cutoff compared to the cutoff used in the manuscript (50th percentile). The inset (dotted lines) shows a zoom-in to the comparison between IMR90 (left) and K562 (right) at the 65th percentile cutoff. Here, for resampling 200 single-cell structures are selected randomly each time. (PNG) [file pcbi.1010392.s008.png]

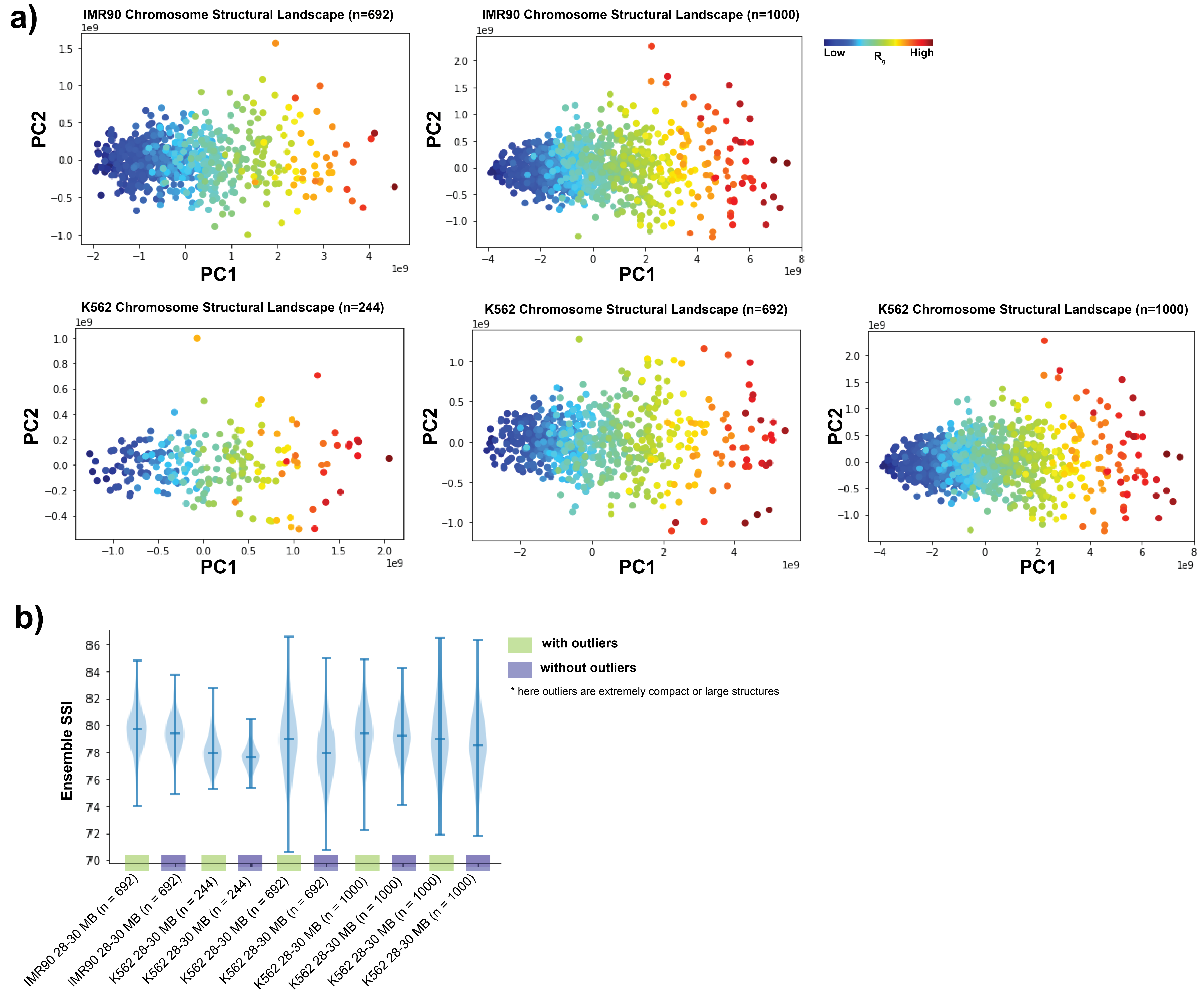

Supplement: S7 Fig — a) Chromosome structural landscape of IMR90 and K562 chr21:28–30 Mb chromatin regions constructed for different numbers of cells after removing extremely compact or large conformations. Points are colored according to the radius of gyration (Rg) of the corresponding structures (dark blue–smaller Rg and dark red–larger Rg). b) Chromosome structural ensemble SSI distributions for chr21:28–30 Mb segment from IMR90 and K562 for different numbers of cells, before (green) or after (blue) removing extremely compact or large conformations (“outliers”). 200 single-cell structures are selected randomly each time for resampling. (PNG) [file pcbi.1010392.s009.png]

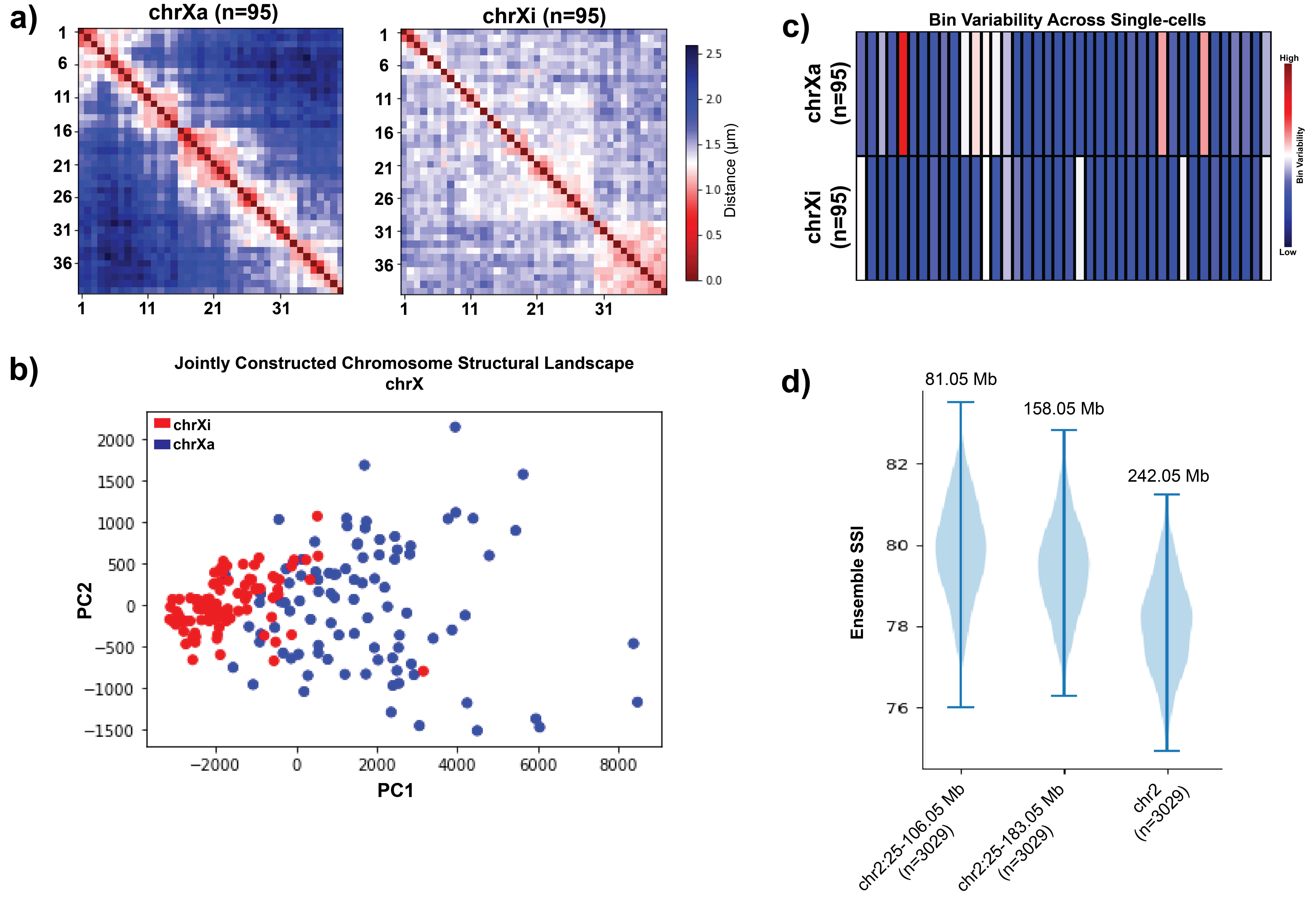

Supplement: S8 Fig — a) Population-averaged distance maps of active and inactive chrX from IMR90 cell at TAD resolution. b) Jointly constructed chromosome structural landscape of active and inactive chrX from IMR90 cell. c) Bin Variability (BSI standard deviation) of active and inactive chrX, where a higher value represents a higher level of structure variation for that bin across the population and vice versa. d) Chromosome structural ensemble SSI distributions for chr2 segments of varying length. Here, for resampling 300 single-cell structures are selected randomly each time. (PNG) [file pcbi.1010392.s010.png]
