# Supplementary material for: Characterizing the variation in chromosome structure ensembles in the context of the nuclear microenvironment
Source: PLoS Comput Biol. 2022 Aug 15;18(8):e1010392. doi: 10.1371/journal.pcbi.1010392 (PMC9410561; doi:10.1371/journal.pcbi.1010392)
Supplement: Attachment — Submitted filename: PloSCB_R2R_July2022.docx [file pcbi.1010392.s013.docx]

**Response to Reviewers**

We appreciate the constructive feedback from the reviewers. Below, we present our point-by-point response to the reviewer comments and concerns.

**Reviewer #1:** In this paper, the authors designed a statistical analysis technique to analyze single-cell chromosome imaging data. I found the results interesting and the analysis technically sound, so I support its publication as is.

We are glad this reviewer supports the publication of the work.

**Reviewer #2:** Identifying and characterizing variability within a large set of genomic structures and understanding its origins and mechanisms is a long-standing and important challenge for genome organization. Das et al. develop a method to approach this question quantitatively. The authors develop a pipeline for analyzing ensembles of single-cell chromosome structure data. The purpose is to characterize the hetero- or homogeneity of the structural ensemble, characterize the variability of distances between different individual genomic loci, and begin to identify the origins of the variability within the ensemble. They analyze data from recent imaging experiments, which mapped distances between genomic loci along several chromosomes / chromosomal regions. They find that the compaction/size of the chromosome is the main mode of variability, but factors such as epigenetic state and the presence and positions of genomic boundaries lead to genomic structural variability between cells too. Furthermore, the overall distribution of observed structures is altered by factors such as cell type and cell cycle phase.

The manuscript is generally well written, although there are some places where the methodology of the calculation is difficult to understand. The analysis generally supports the conclusions, except for a few points that could be refined to more narrowly state the conclusion. My main questions regard the robustness of the method (across repeat experiments, different genomic regions, and changes in thresholds applied in the analysis), requirements (in terms of number and quality of samples), and the interpretation of the results (in terms of the meaning of the differences in the “structural similarity index”, etc.). Another issue is how this method might be used to identify new properties of chromosome structure or whether it is better suited for providing quantitative/statistical support for existing hypotheses. These questions should be addressed in the revision. Nonetheless, I think this manuscript is an interesting and useful contribution to the existing literature on genome structure and its analysis, and I would support of acceptance with revisions addressing my comments below.

1. The description of pairwise structure-structure similarity matrix is difficult to understand. After several re-readings of the relevant paragraph, I can see that all of the elements of the calculation are present, but a few extra words to be clear that, for example, each dot product is a single number from the product of two matrices that is then put into another matrix (on which PCC will be calculated, etc.), among other steps in the computation, would be helpful.

We have now clarified our description of this method—see lines 180-187

2. The main metric, SSI, is difficult to interpret. Because SSI is scaled into arbitrary units, it mainly gives a qualitative comparison between different datasets despite being a calculable quantity. But it is unclear what the size of the effects are. What does a 10% difference in SSI mean for the distribution of single-cell structures? It might be helpful to measure SSI for some model structures such as a (confined) Rouse polymer or a highly crosslinked chain.

We have now clarified in our methods section and figure labeling exactly how the SSI metric is calculated. While we call this “arbitrary units”, the quantitative value is still directly linked to the network entropy calculation and therefore the scale is consistent across all figures, rather than being arbitrarily expanded (as would happen in a min-max scaling, for example).

The nature of the SSI calculation means that simulated fluctuations around a single structure (highly crosslinked chain) will have a high SSI because the structure will be sampling a landscape of structures that are all closely related to each other and are thus highly connected to each other in network space. Low SSI results when there are multiple different possible structures that are more distinct from each other. This only happens when other constraints are added, such as the epigenetic state interactions that we modeled in our polymer simulations. Overall, a simple Rouse model or random polymer limit is too different from the biologically relevant systems we are considering to provide a good estimate of a relevant SSI range.

Instead, we note several comparisons in the manuscript that indicate the biological relevance and meaning of the amount of change in the SSI metric. We note that a 10% change in SSI (78-88 for example) is typical of both numerous comparisons across different real biological regions and also the amount of SSI change observed with simulated differences in epigenetic state. The most dramatic SSI difference we detect is between Xa and Xi, which are known very different structural states. This leads us to think that a 12% change in SSI (as seen in this case) is a biologically significant amount of difference. In our new analysis of sliding windows across chromosome 21, we see about a 20% change in SSI across different chromosome windows.

We have also examined subsampled groups of structures (highlighted red) that give different SSI values to demonstrate how a certain amount of difference in SSI relates to clustering vs. sparsity in the structure landscape. Below, you can see that to achieve an SSI of 40, the individual structures would have to be very sparse, and we have never seen this level of disconnectedness in real biological data. Thus, we conclude that the range of values we report (SSI 60-95) is the extent of the biologically relevant range and can be interpreted as such.

SSI = 40:

SSI = 75:

3. The authors refer to a pairwise structure-structure “similarity” matrix constructed from dot products of median-centered distance matrices. “Similarity” instead of “correlation” seems slightly misleading to me here. Further, it may be better to rescale the dot products by the product of the mean median-centered distance of each structure (in the same way that correlations of fluctuations in physical systems are typically normalized). This would avoid the following problematic scenario: consider two distance matrices identically equal to the median matrix; they would have a similarity of zero despite being the same (since the median-centered matrices would be identically zero).

We agree that the term “similarity matrix” is not accurate here, for the reasons described. We have instead revised this to “structure comparison matrix”. We have left the calculation as stated for the following reason—as when protein structure analyses measure the deviation from the mean structure position, we want to measure the deviation of each chromosome structure from the median distance and then compare these deviations. We choose not to rescale or normalize because we want to preserve the amplitude differences in the fluctuations in structure. This is analogous to the fact that many PCA analyses of structural ensembles are based on the covariance matrix, which preserves such differences in amplitude, rather than the correlation matrix, which would normalize away these differences. Another way of saying this is that we would like to the version without normalization and keep the amplitude difference as an meaning difference that reflects flexibility difference of the biopolymers. Indeed, most PCA analysis performed on structural ensembles is based on covariance matrix, not correlation matrix (the normalized version of covariance matrix).

4. The analysis could be improved if the authors could be more systematic about the trends of SSI with variables such as number of cohesin or compartment boundaries and variability of epigenetic states. As it stands, a small number of examples are compared, and plausible explanations are given, but it is unclear how well these trends and explanations hold up over larger chromosomal regions, more genomic regions, or even the entire genome.

To address this, we have now calculated the SSI systematically for all windows across chromosome 21 and have demonstrated that the SSI correlates well with the number of boundaries per window (New fig S9d,e). We note that it is not possible for us to perform this calculation genome-wide, because high resolution data is only available across certain chromosomes (chr21 and chr2) (from Su et al 2020).

5. The authors state that higher uniformity of nucleolus association leads to higher continuity of states at the structural ensemble level. But how do we know the direction of causality; could the structure of ensemble-space instead facilitate more consistent nucleolus association? More generally, can the direction of causality be established within this pipeline for phenomena that are not already (somewhat) well understood, such as loop extrusion and compartments/polymer-polymer phase separation?

We agree that our initial language can be improved and have thus clarified this point in our revision.  It is true that we cannot know for sure whether it is nucleolus association or some other factor that leads to altered SSI, and we have adjusted our language to clarify that we are noting a relationship between biological features, such as nucleolus association and structural landscape continuity. The “direction of causality” does not seem like quite the right concept here, because nucleolus association is a physical state while the landscape feature is a mathematical description/manifest. These are different types of things, and a mathematical description cannot cause a physical state. But, it is correct that there could be some other factor that leads to the changes in SSI and that this in turn influences nucleolus association. On the other hand, our polymer simulation can directly test how different factors/constraints could contribute to the types of structural heterogeneity we observe. We used these simulations to demonstrate that differential interactions between different epigenetic states can cause different patterns of structural variations. This doesn’t definitively prove that this is the underlying biological cause, but it lends some more creedence to a suggestion of causality.

6. How many single-celled structures are needed to perform this analysis effectively, and for example, reliably compute SSI?

This is difficult to answer definitively. The answer is in many ways like any other question of sampling and power to detect differences. As you can see in the figure below, if we use fewer structures (with resampling size = n/2 in each case), the distribution of SSI values becomes larger. This would make it more difficult to reliably detect a small difference between structural landscapes. The fewer structures there are, the more likely it is for the landscape to look artificially very discontinuous (see the example above in answer to question #2 of SSI = 40 with small sample size of 20 structures out of the total number actually measured). However, we note that the direction of difference between IMR90 and K562 is the same in all the sample sizes and the variance in SSI at n=100 for IMR90 is almost the same as for n=200. This suggests that the relative comparisons of the metric are robust to changes in sample size, but at some point (less than ~50 structures) the variability becomes too high to confidently characterize the SSI.

7. How sensitive are calculations and analysis to thresholds used for distance and correlation matrices?

The spatial distance thresholds we use are derived from previously published values. We have now examined the impact of thresholds for the correlation matrix, with the results shown below and in Figure S6c. Here, we show the ensemble SSI results for a lower (35^th^ percentile) and higher (65^th^ percentile) correlation matrix cutoff compared to the one used in the manuscript (50^th^ percentile). The inset shows a zoom in to the comparison between IMR90 (left) and K562 (right) at the 65^th^ percentile cutoff.

Using a cutoff lower than the 50^th^ percentile used in our manuscript shows a similar relative comparison between IMR90 and K562. But using a higher cutoff can result in many disconnected subnetworks which may affect the conclusions from the SSI calculation. In the case of disconnected networks, each of the independent connected components act as a highly similar network within itself, and as a result, each component produces higher entropy values which leads to an overall high ensemble SSI score of the system. On the other hand, having a single connected network represents the connection between both the similar and dissimilar structures in the system and produces reliable ensemble SSI scores of the system. For example, in the above figure for 65^th^ percentile cutoff, K562 produces a higher ensemble SSI score compared to IMR90 in comparison to other two cutoffs. As the K562 structural ensemble is more heterogeneous than the IMR90, with higher cutoff the K562 network becomes disconnected. At that point, the heterogeneity of the network is no longer captured by the calculation, but instead the highly connected nature of each subcomponent dominates the result. Using a lower cutoff tends to maintain the overall relative measurements of landscape connectivity, but adds more non-significant edges to the calculation, decreasing the precision of the result. Therefore, in this work we decided to set the cutoff to 50^th^ percentile, a balance between high and low cutoff. This discussion and result is now included in the Results section and Fig S6.

8. In several places, the authors refer to evaluating “dynamics” or contributions (e.g., of epigenetics) to dynamics, but the data is an ensemble of snapshots from different single cells; therefore, dynamics can at best be inferred from the analysis of the structure ensemble.

It is correct that we are always seeing the heterogeneity across single cell snapshots, which can results from both dynamics and stable heterogeneity. For this reason, we carefully avoid referring to the fluctuations as dynamics alone. We have further clarified any instance where we refer to dynamics to ensure we do not claim to measure dynamics directly.

The only remaining places where we use the term “dynamics” refer to things where we really mean dynamics (such as “loop extrusion dynamics”) or where we are suggesting a possible connection to previous work (“Our observations are consistent with the previously published idea that cohesin loop extrusion acts to increase chromosome conformation dynamics, promoting distal contacts and countering the tendency of domains to settle into more discrete sub-structures (61,62)”)

9. It would be useful for the authors to include a short explanation of the meaning and use of the Shannon-Jayne entropy for context, especially since the main metric studied in the manuscript relies on this entropy.

We have added this explanation, along with citation of other common uses of this type of metric in biological analysis, to lines 209-218.

10. The conclusions about the heterogeneity of regions with less uniform epigenetic marks are interesting. Can the authors comment on whether this is in line with expectations from polymer physics for heteropolymers?

Our simulation of polymers with different interactions based on different simulated epigenetic states is indeed a test of what would be theoretically expected for such a heteropolymer.

11. The low resolution of the figures (particularly figure 5) and the small label sizes on the axes of the matrices in Figs. 1b,c make the figures difficult to read.

We have now improved the resolution and label size on the figures in the provided high resolution .tiff versions.

12. Fig 1b panel 3 says mean-centered instead of median-centered.

We appreciate this catch and have corrected this typo.
